# Gelatinase B/Matrix Metalloproteinase-9 as Innate Immune Effector Molecule in Achalasia

**DOI:** 10.1038/s41424-018-0076-6

**Published:** 2018-11-19

**Authors:** Janette Furuzawa-Carballeda, Lise Boon, Gonzalo Torres-Villalobos, Fernanda Romero-Hernández, Estefania Ugarte-Berzal, Erik Martens, Jennifer Vandooren, Vasily Rybakin, Enrique Coss-Adame, Miguel Valdovinos, David Velazquez-Fernández, Ghislain Opdenakker

**Affiliations:** 1 Department of Immunology and Rheumatology, Instituto Nacional de Ciencias Médicas y Nutrición Salvador Zubirán, Mexico City, Mexico; 2 Department of Microbiology and Immunology, Laboratory of Immunobiology, Rega Institute for Medical Research, KU Leuven, University of Leuven, Leuven, Belgium; 3 Department of Experimental Surgery, Instituto Nacional de Ciencias Médicas y Nutrición Salvador Zubirán, Mexico City, Mexico; 4 Department of Surgery, Instituto Nacional de Ciencias Médicas y Nutrición Salvador Zubirán, Mexico City, Mexico; 5 Department of Gastroenterology, Instituto Nacional de Ciencias Médicas y Nutrición Salvador Zubirán, Mexico City, Mexico

## Abstract

**Objectives:**

Achalasia is a primary esophageal motility disorder resulting from selective loss of inhibitory neurons in the esophageal myenteric plexus, likely due to an autoimmune response with involvement of the adaptive immune system. Innate immune processes of the host constitute the bridge between environmental etiological factors and the adaptive immune system. Although these remain poorly investigated, they might be of diagnostic and therapeutic relevance. In view of the role of extracellular proteolysis in organ-specific autoimmunity, we studied gelatinases of the matrix metalloproteinase (MMP) family in achalasia patients.

**Methods:**

The presence of MMP-2 and MMP-9 proteoforms was analyzed in sera of two cohorts of achalasia patients. Additionally, with the use of immunohistopathological analysis, in situ MMP-2 and MMP-9 expression was investigated. Finally, we tested the paradigm of remnant epitopes generating autoimmunity (REGA) for achalasia-associated autoantigens by evaluating whether autoantigenic proteins are cleaved by MMP-9 into remnant epitopes.

**Results:**

We showed significantly increased ratios of MMP-9/MMP-2 and activated MMP-9/proMMP-9 in sera of achalasia patients (*n* = 88) versus controls (*n* = 60). MMP-9-positive and MMP-2-positive cells were more abundant in achalasia (*n* = 49) versus control biopsies from transplant donors (*n* = 10). Furthermore, extensive damage within the plexus was found in the tissues with more MMP-9-positive cells. Additionally, we documented achalasia-associated autoantigens PNMA2, Ri, GAD65, and VIP as novel MMP-9 substrates.

**Conclusions:**

We provide new biomarkers and insights into innate immune mechanisms in the autoimmune pathology of achalasia. Our results imply that extracellular protease inhibition is worthwhile to test as therapeutic intervention in achalasia.

## Introduction

Achalasia is a primary esophageal motility disorder with an incidence of about 1 in 100,000 persons annually^1,2^. The disease is defined by absent peristalsis of the esophagus and failure of lower esophageal sphincter relaxation upon swallowing resulting in severe dysphagia, regurgitation, aspiration, chest pain, and weight loss. Achalasia results from a selective loss of inhibitory neurons in the esophageal myenteric plexus, most likely due to an autoimmune response with involvement of adaptive immune cells and molecules. Like other tissue-specific autoimmune diseases, such as multiple sclerosis, arthritis, and diabetes, achalasia is suggested to be a T lymphocyte-mediated disease in which autoantigenic peptides (re)activate T cells leading to imbalances in cytokine profiles and inflammation^3,4^. Because no animal model exists, medical progress completely depends on comparisons of patient cohorts and on clinical research by the grace of consenting volunteers who donate tissues. So far, the only efficient therapies are myotomy of the lower esophageal segment and PerOral Endoscopic Myotomy (POEM)^5^ and endoscopic pneumodilatation as a relief of dysphagia in this severe swallowing disorder^6^.

The susceptibility to develop autoimmune achalasia is based on the genetic background of the host and a major contributor to this susceptibility is the human leukocyte antigen (HLA) gene cluster on chromosome 6, encoding the major histocompatibility complex molecules (MHC class I and II). Determinant genetic disease association markers for achalasia are class II HLA-DQβ1 (HLA-DQβ1*03:04, HLA-DQβ1*05:03, HLA-DQβ1*06:01 and DQβ1*0602), HLA-DQα1 (HLA-DQα1*01:03) and HLA-DRβ*12^7–9^. Processing of (auto)antigens is intrinsically an intracellular process executed by the proteasome for presentation in MHC I (HLA-A, HLA-B, HLA-C) and by lysosomal enzymes for presentation in MHC II (HLA-DP, HLA-DQ, HLA-DR). Additionally, a single nucleotide polymorphism at the lymphotoxin-α and tumor necrosis factor-α locus was suggested to be a susceptibility factor for idiopathic achalasia^10^. More recently, innate immune cells and extracellular proteolysis as triggering steps were added to the classical paradigms of autoimmunity^11,12^, but whether this paradigm is operating in achalasia remained unknown. One enzyme of extracellular proteolysis is of particular interest in inflammatory and autoimmune diseases: gelatinase B or matrix metalloproteinase-9 (MMP-9).

One of the potential environmental triggers for the development of immune-mediated diseases are infections. In patients with achalasia, infection with neurotropic viruses, most importantly herpes simplex virus type 1 is thought to be a major determinant in the development of the disease^13,14^. In patients with multiple sclerosis, another organ-specific autoimmune disease, vitamin D has been demonstrated as a protective factor and the link with latitude (and UV-light radiation) as an environmental factor has been made^15–17^. In line with these studies on multiple sclerosis, Becker and colleagues suggest that a geospatial north–south gradient variation prevalence of achalasia may exist due to genetic variations^9^.

Environmental triggers, including herpesvirus infections in individuals with a genetic background, susceptible to develop an autoimmune response, may induce inflammatory cytokines and chemokines that attract and activate myeloid cells^4,11^. This may lead to disturbances in the local extracellular protease balances – the net result of activated proteases and their inhibitors – resulting in the generation of remnant epitopes, subsequent cellular uptake and intracellular processing and MHC II presentation of autoantigens. We coined this paradigm the REGA model (for “remnant epitopes generate autoimmunity”) to address extracellular cytokine-regulated proteolysis and myeloid cells as important players in the early phases of autoimmune reactions^11,12^. Extracellular proteolysis was related with and tested for the inflammation-associated protease gelatinase B/MMP-9 in the following diseases: multiple sclerosis, rheumatoid arthritis and diabetes^11,12^. Furthermore, these insights on extracellular proteolysis improved the early diagnosis and treatment of multiple sclerosis^18,19^.

Recently, the presence of MMP-9 in achalasia was described^4^. However, neither its activity in the pathogenesis of achalasia, nor processing of relevant substrates are known. Here we provide translational data about the role of MMPs, specifically about the gelatinases, in achalasia pathogenesis. The data suggest that the ratio MMP-9/MMP-2 might be useful as novel serum marker for achalasia. In this study, we demonstrated that in situ MMP-9 levels are correlated with the Chicago classification of achalasia^20^. Finally, we document that MMP-9 cleaves a number of known and novel achalasia-associated autoantigens into remnant epitopes and therefore might be a major player during the pathogenesis of achalasia.

## Methods

### Patients

One hundred two patients with achalasia (type I *n* = 24; type II *n* = 73; type III *n* = 5) were enrolled and serum and tissue samples were used for zymography analysis, immunohistochemistry and tissue cleavage experiments. The diagnosis of achalasia was based on clinical evaluations as well as on oesophagram, high-resolution manometry^21^ (classified based on Chicago v3.0)^20^ and endoscopy results. Patients were excluded from study participation according to diagnosis of secondary achalasia due to Chagas disease, esophageal stricture, gastric, esophageal cancer or esophageal scleroderma, human immunodeficiency virus (HIV), or hepatitis C virus (HCV) infections. Peripheral blood samples were obtained from age-matched healthy blood donors who volunteered at the Instituto Nacional de Ciencias Médicas y Nutrición Salvador Zubirán blood bank. Healthy donors were also interviewed to exclude any disease, including known cardiovascular, metabolic, inflammatory, neoplastic or autoimmune diseases, use of prednisone or other immunosuppressive drugs and concurrent infections. None of the healthy volunteers had serum antinuclear antibodies (ANAs). Demographic, clinical and laboratory information was collected (Table 1). For the first serum marker study 38 samples of achalasia (type I *n* = 9; type II *n* = 27; type III *n* = 2) were compared with sera from controls (*n* = 33). These samples were collected between January 2013 and October 2015. For the second serum validation and histopathology studies, another sample set of 50 patients with idiopathic achalasia (type I *n* = 10; type II *n* = 37; type III *n* = 3) was obtained between January 2016 and March 2017. Information about the medical records of the cohort of achalasia patients is provided in Table 1. Lower esophageal sphincter muscle samples from organ donors for transplantation (transplant donors (TD), *n* = 10) were included as tissue controls. None of the transplant donors had previously known metabolic, inflammatory, neoplastic or autoimmune diseases. No viral infections with cytomegalovirus (CMV), hepatitis C virus (HCV), hepatitis B virus (HBV), and human immunodeficiency virus (HIV) were detected. Tests for syphilis and serum ANAs were negative. The cause of death in 6 of the donors was by brain injury, 2 by subarachnoid hemorrhage, and 2 by hemorrhagic stroke. Demographic, clinical, and laboratory information was also collected (Table 1). All patients and tissue donors were diagnosed and recruited from the Outpatient Clinics of Gastroenterology and Surgery of the Instituto Nacional de Ciencias Médicas y Nutrición Salvador Zubirán (a tertiary referral center in Mexico City, Mexico). All patients and control donors provided written informed consent and all sampling was done under the tenets of the Declaration of Helsinki, 1989, for research use of human material. The protocol was approved by the Ethical Medical Committee of Instituto Nacional de Ciencias Médicas y Nutrición Salvador Zubirán.

**Table 1:** Demographic, clinical, and laboratory variables of achalasia cohorts

	Type I Achalasia (*n* = 24)	Type II Achalasia (*n* = 73)	Type III Achalasia (*n* = 5)	Tissue donor (*n* = 10)	Healthy blood donor (*n* = 97)
Sample (sera / tissue)	✓ / ✓	✓ / ✓	✓ / ✓	− / ✓	✓ / −
*Demographics*					
Age (years)					
Mean ± SD	40.9 ± 16.2	41.1 ± 14.9	53.2 ± 15.5	34.6 ± 12.6	43.5 ± 14.0
Median	38.5	40.0	44.0	33.0	44.0
Range	18–79	17–77	41–77	15–56	21–76
Sex					
Female/male	12 / 12	51 / 22	3 / 2	4 / 6	68 / 29
(%)	50 / 50	70 / 30	60 / 40	35 / 65	70 / 30
Disease evolution (months)					
Mean ± SE	31.6 ± 6.6	19.8 ± 2.9	58.8 ± 45.4	NA	NA
Median	18.0	12.0	12.0		
Range	5–150	1–120	6–240		
*Clinical variables*					
Dysphagia, *n* (%)	23 (96)	73 (100)	5 (100)	ND	0 (0)
Regurgitation, *n* (%)	21 (88)	69 (95)	3 (60)	ND	7 (7)
Weight loss, *n* (%)	11 (46)	68 (93)	4 (80)	ND	1 (1)
Heartburn, *n* (%)	17 (71)	52 (71)	1 (20)	ND	6 (6)
Other autoimmune disease, *n* (%)	3 (13)	11 (15)	2 (40)	0 (0)	0 (0)
Allergy and asthma, *n* (%)	3 (13)	13 (18)	0 (0)	2 (2)	5 (5)
*Viral exanthematous childhood disease*					
Chickenpox, *n* (%)	18 (75)	49 (67)	2 (40)	ND	ND
Measles, *n* (%)	12 (50)	22 (30)	1 (20)	ND	ND
Rubella, *n* (%)	1 (4)	6 (8)	0 (0)	ND	ND
Hepatitis, *n* (%)	1 (4)	4 (6)	0 (0)	ND	ND
Mumps, *n* (%)	0 (0)	3 (4)	0 (0)	ND	ND
*Environmental exposure*					
Tobacco smoke exposure, *n* (%)	7 (29)	24 (32)	2 (40)	ND	3 (3)
Biomass smoke exposure, n (%)	5 (21)	17 (23)	2 (40)	ND	1 (1)
*Laboratory data*					
Anti-nuclear antibodies (%)	37	28	80	0	1
**Immunohistochemistry**	**Type I Achalasia (** ***n*** ** = 15)**	**Type II Achalasia (** ***n*** ** = 31)**	**Type III Achalasia (** ***n*** ** = 5)**	**Transplant donor (** ***n*** ** = 10)**	
*MMP-9 (% positive cells per field)*					
Mean ± SE	6.5 ± 0.8	8.0 ± 1.0	16.7 ± 3.5	0.1 ± 0.1	
Median	6.0	9.0	18.0	0.0	
Range	0–10.5	2–20	10–22	0–0.5	
*MMP2 (% positive cells per field)*					
Mean ± SE	1.4 ± 0.5	3.7 ± 0.6	2.3 ± 1.2	0.9 ± 0.4	
Median	0.0	2.0	3.0	0.5	
Range	0–5	0–10	0–4	0–3.5	

*SD* standard deviation, *SE* standard error, *NA* not applicable, *ND* not determined; *P < 0.05

### Tissue biopsies

Biopsies of achalasia patients were taken during Heller myotomy. After the myotomy was completed with no use of energy devices, a full thickness muscle biopsy, 2 mm wide and 2 cm long, was obtained by cutting with scissors and was immediately preserved. For the healthy tissue controls, patients for organ donation were included. The esophago-gastric junction was obtained during organ procuration, with previous signed informed consent from the family. The esophago-gastric junction with 3 cm of esophagus and 2 cm of stomach was taken. The tissue was transported at 4 °C in Bretschneider’s (Custodiol) solution in a period of 4–6 h. Subsequently, a full thickness biopsy of the muscle (thus including the myenteric plexus) of the esophagus was obtained. Tissue was immediately formalin-fixed, and paraffin-embedded.

### High resolution esophageal manometry protocol

A high resolution esophageal manometry (HRM) was performed in every patient at baseline and before being referred to surgery. A solid-state HRM probe with 36 circumferential sensors was used (Given Imaging, Yokneam, Israel). Having the patient in a sitting position and at 45°, stationary HMR was performed, as follows. After a 12 h fasting period, the probe was inserted transnasally until passing the esophagogastric junction and assessed visually on the computer screen of the imaging device. Ten water swallows of about 5 mL, separated by 30 s were provided. Analyses were performed using Manoview 2.0 (Given Imaging) and achalasia patients were classified according to the latest Chicago classification^20^ into three groups: (i) type I achalasia (without pressurization within the esophageal body), (ii) type II (with pan-pressurization), and (iii) type III (spastic). Classification was performed by two gastroenterologists, both being experts in high-resolution esophageal manometry.

### Zymography analysis

**Fig. 1:**
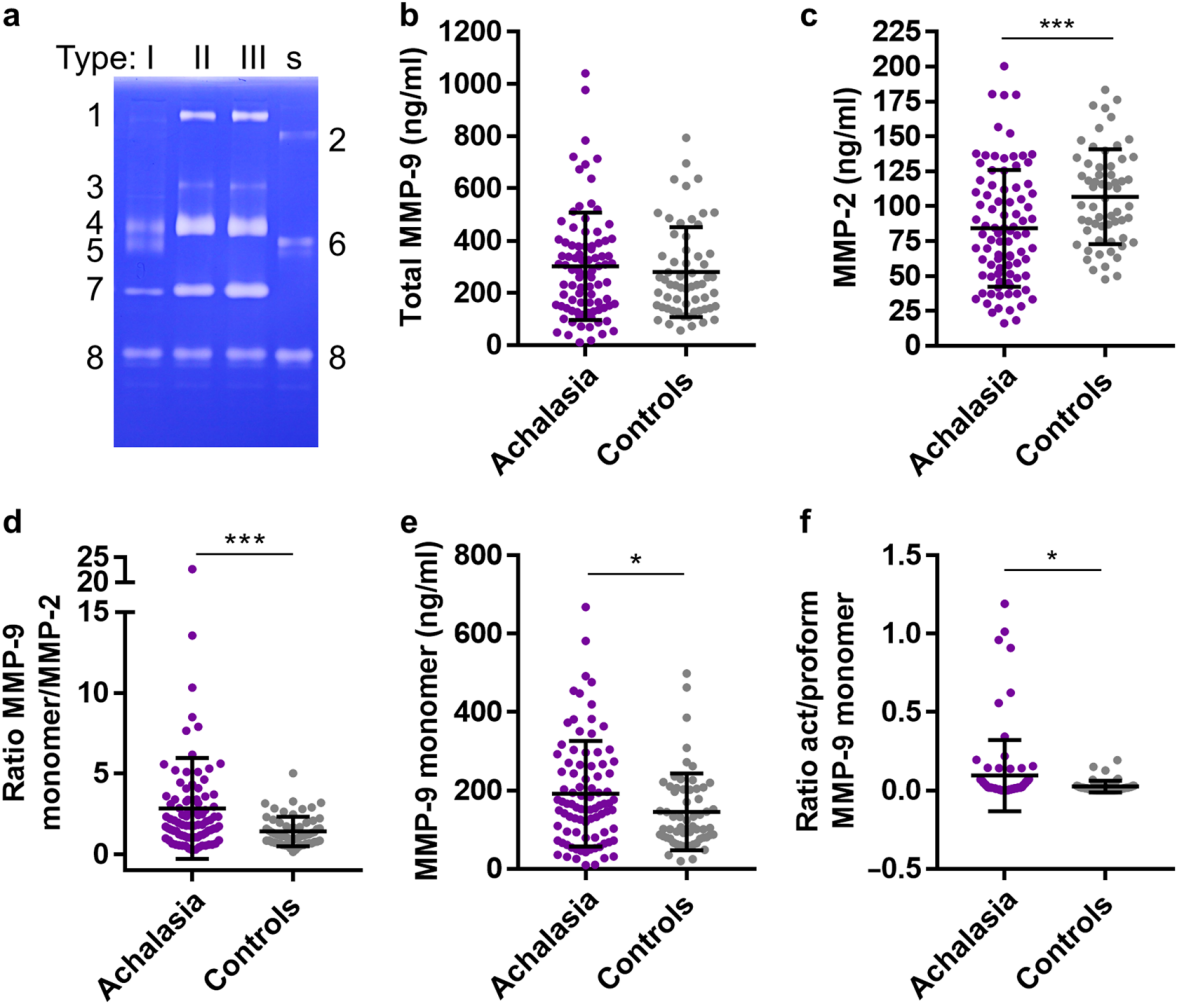
Quantitative gelatin zymography analysis of achalasia patient sera. Serum samples from patients with achalasia (*n* = 88) and controls (*n* = 60) were spiked with a known amount of a recombinant deletion mutant of human gelatinase B, pre-purified by gelatin-Sepharose affinity chromatography and subjected to zymography analysis. **a** Key for the interpretation of the gelatin-zymolysis. On top the type of achalasia patients from whom sera were analyzed are indicated, whereas the letter s indicates the standard sample with three recombinant human gelatinase B/MMP-9 forms. At the left side the numbers indicate gelatinase forms as follows: 1 = trimer form of MMP-9; 3 = covalent complex between MMP-9 and neutrophil gelatinase B-associated lipocalin (NGAL); 4 = proform of MMP-9; 5 = activated form of MMP-9; 7 = gelatinase A/MMP-2; 8 = recombinant deletion mutant spiked into each sample. At the right side a standard mixture of recombinant molecules is included as follows: 2 = recombinant trimer MMP-9; 6 = recombinant monomer proMMP-9; 8 = recombinant mutant of human MMP-9 with deletion of the O-glycosylated and hemopexin domains. **b** Total MMP-9 levels in achalasia versus control sera. **c** Levels of MMP-2 in achalasia versus control. **d** Ratios of MMP-9 monomers and MMP-2 in the comparisons of sera from achalasia patients and controls. **e** Levels of monomer proMMP-9 in achalasia versus control sera. **f** Ratios of monomeric activated MMP-9 and proMMP-9 in the comparisons of achalasia and control sera. For all cohorts the mean levels ±SD are provided, *p < 0.05 and ***p < 0.001. Achalasia: n = 88. Controls: n = 60

The analysis of gelatinases in serum samples and esophageal biopsy materials was done at the Rega Institute, University of Leuven. For all samples, we first applied a prepurification step on gelatin-Sepharose beads^22^. Although this procedure takes extra time, it reduces significantly background signals and yields better resolution for determination of molecular forms (because in the presence of albumin and other bulk serum proteins the electrophoretic mobility of gelatinases is severely distorted). With the inclusion of spiking and an internal standard preparation containing recombinant MMP-9, this procedure makes quantitative zymography possible^23^. Therefore, all samples were spiked, prior to prepurification, with a known amount of a recombinant deletion mutant of human MMP-9 that lacks both the O-glycosylated and the hemopexin domains^24^. On all zymography gels we included a recombinant standard mixture of three recombinant proteoforms of human MMP-9, as produced in Sf9 insect cells: trimeric MMP-9, monomeric MMP-9, and the above mentioned deletion mutant. Zymogram examples are provided in the Supplementary Figure 1 and the key to define the various gelatinase forms in the standard mixture as well as in human sera is shown as Fig. 1a.

### Double-staining procedure

In order to determine the MMP-9-positive and MMP-2-positive immunoreactive cells, 5 µm-thick sections of formalin-fixed and paraffin-embedded tissue were placed on positively charged slides. Sections were deparaffinized and rehydrated through a series of xylene and graded alcohols. Enzyme antigen retrieval was carried out during 2 min (Enzo Life Sciences, Inc., Farmingdale, NY, USA) and tissues were blocked with 3% H_2_O_2_. Then non-specific background staining was avoided with the immunohistochemistry background blocker (Enzo Life Sciences). We included esophagus tissue samples from transplant donors as extra control tissues (Table 1). MMP-9- and MMP-2-producing cells were evaluated with the use of a Multiview (mouse-HRP/rabbit-AP) immunohistochemistry kit (Enzo Life Sciences, Inc., Farmingdale, NY, USA). This procedure is a sequential double staining method in which the rabbit polyclonal anti-MMP-2 IgG (Santa Cruz Biotechnology, CA, USA) and mouse monoclonal anti-MMP-9 IgG1 antibody (REGA-2D9) were incubated during 40 min at room temperature. Slides were washed and then incubated with PolyView IHCh reagent (anti-mouse-HRP) and PolyView IHCh reagent (anti-rabbit-AP) for 20 min. Finally, MMP-9 antigens were visualized using horseradish peroxidase (HRP)/3,3′-diaminobenzidine (DAB) and the MMP-2 antigens with alkaline phosphatase (AP)/Permanent Red. Tissues were counterstained with Mayer’s hematoxylin and mounted in aqueous mounting medium. Negative control staining was performed with the universal negative control reagent specifically designed to work with rabbit, mouse, and goat antibodies (IHCh universal negative control reagent, Enzo Life Sciences, Inc.) The blank was incubated with phosphate buffered saline-egg albumin (SIGMA-Aldrich) instead of the primary antibody. Both controls satisfactorily excluded nonspecific staining and endogenous enzymatic activities. MMP-9- and MMP-2-expressing cells were counted in at least three optical fields from each slide in 320× high power magnifications. The average values per slide were used for statistical analysis. Results were expressed as the mean ± standard error of the mean (SEM) of cells quantified by the program Image Pro-Plus version 5.1.1.

### Cleavage experiments

The following proteins were found to be of sufficient purity for cleavage experiments and purchased from the respective commercial providers: Recoverin (GENTAUR Europe, Voortstraat 49 1910 Kampenhout Belgium), vasoactive intestinal protein (VIP, GENTAUR Europe), paraneoplastic antigen MA2 (PNMA2, NOVUS Biologicals, 19 Barton Lane Abingdon, OX14 3NB, United Kingdom), Ri protein (NOVUS Biologicals) and glutamic acid decarboxylase of 65 kilodalton (GAD65 from mouse brain tissue, prepared in-house). PNMA2 (1 µM), VIP (10 µM), Ri (2 µM) and Recoverin (10 µM) were incubated alone or with cdMMP-3-activated MMP-9 at different enzyme substrate ratios (1/10, 1/100, 1/1000 and 1/10 000). Aliquots were taken at several time points (0, 5, 10, 30, 90, 180, 420 minutes and overnight). Incubations were done at 37 °C in 50 mM Tris pH 7.4, 150 mM NaCl, 5 mM CaCl_2_, 0.01% Tween 20. Samples were separated by SDS-PAGE and protein staining was done using the Silver Stain procedure, following the manufacturer’s procedure (Pierce Silver Stain Kit, Thermo Fisher Scientific).

### Statistical analysis

Descriptive statistics were performed for the immunohistopathological analysis, and categorical variables were compared using the *Χ*^2^ test or Fisher’s exact test. One-way analysis of variance on ranks by Holm–Sidak method and Dunn’s test was performed for all pairwise multiple comparison procedures. Statistical analysis was done using the Sigma Stat 11.2 program (Aspire Software International, Leesburg, VA, USA). Data were expressed as the median, range, and mean ± SD/standard error of the mean (SEM). Non parametric testing (Kruskal–Wallis test) was done to analyze MMP-2 and MMP-9 levels obtained by zymography analysis. The *P* values ≤0.05 were considered as significant. We reported non-parametric correlations using Spearman coefficients among the active/proMMP-9, MMP-9/MMP-2, and total MMP-9 with clinical parameters (overweight, obesity, autoimmunity, tobacco consumption, chickenpox, rubella, measles, hepatitis, mumps, and biomass smoke exposure).

## Results

### Serum gelatinase forms

Previously, MMP-9 and its inhibitor (tissue inhibitor of metalloproteinase-1 or TIMP-1) have been detected by immunohistochemistry in biopsies of achalasia patients^4^. Because the analysis of gelatinases in sera has been found useful as parameter for gastrointestinal inflammation^25^, we first evaluated and compared the levels of all the gelatinase forms in achalasia patients and control serum samples by quantitative zymography analysis. Fig. 1a and Supplemental Fig. 1 show, respectively, the zymography key and primary data of this analysis. The following enzyme forms were detected in human sera: MMP-9 trimers^26^, MMP-9-NGAL complex^23^, proMMP-9, activated MMP-9 and MMP-2, while activated MMP-2 was not detected. When comparing data from the combined two cohorts of achalasia patients (*n* = 88) with matched controls (*n* = 60), we observed that the total levels of MMP-9 (Fig. 1b) were similar in achalasia and controls, whereas the levels of the MMP-9 monomer (Fig. 1e) and MMP-2 levels (Fig. 1c) were significantly altered in achalasia versus control. In many inflammatory diseases, the ratio of MMP-9 versus MMP-2 is a useful marker^23,25,27,28^, as was corroborated here in achalasia versus controls (Fig. 1d). Because we prepurified all samples, the resolution was sufficient to discriminate between the proform and activated forms of the MMP-9 monomer (Supplemental Fig. 1). Although activated forms are less stable and less commonly detected than the proforms, we detected significantly more activated MMP-9 monomers in achalasia than in control sera (Fig. 1f). Individual cohort data are shown in Supplemental Fig. 2.

### Gelatinase ratios and achalasia types

**Fig. 2:**
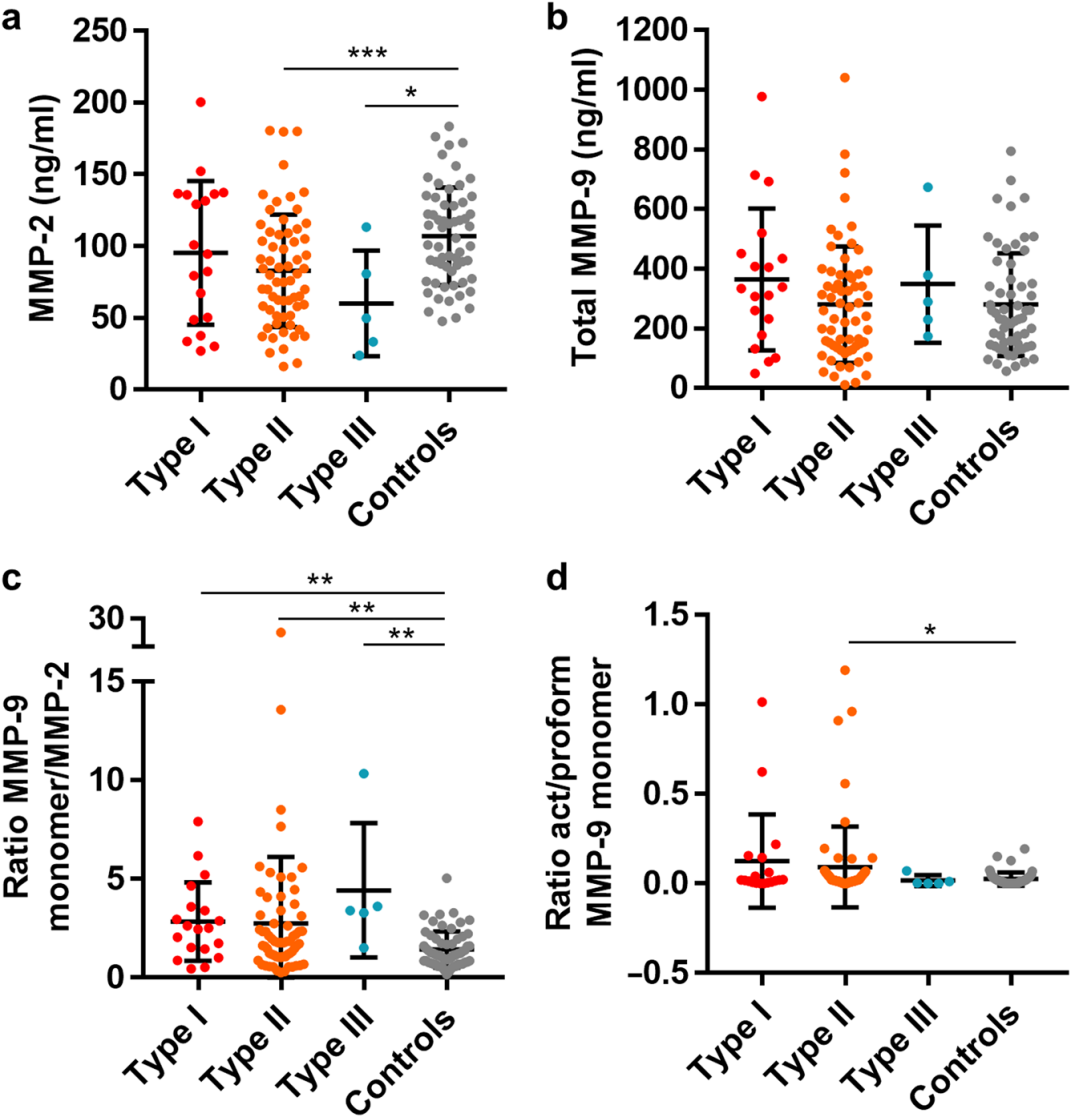
Zymography analysis of achalasia. Patients with defined achalasia type I, type II, type III, and controls were discriminated, and sera were analyzed as indicated in Fig. 1 and Supplemental Fig. 1. **a** MMP-2 levels in the serum of individual patients according to achalasia type. **b** MMP-9 levels in analyzed sera. **c** Ratios of MMP-9 versus MMP-2 regarding to types of achalasia. **d** Ratios of activated versus proMMP-9 monomer forms in the defined types. For all types of achalasia the mean levels ±SD are provided, *p < 0.05; **p < 0.01; ***p < 0.001

As a next step we compared the gelatinase forms according to the Chicago classification of achalasia^20^. The numbers of patients with achalasia type I and III were limited (*n* = 19 and *n* = 5, respectively) in comparison with type II (*n* = 64) and controls (*n* = 60). The ratio of MMP-9/MMP-2 was found to be significantly higher in achalasia type I, II, and III compared to controls (Fig. 2a–c). In the analysis of activated MMP-9 monomers versus the proform of MMP-9 (Fig. 2d) significant differences were found for type II achalasia sera versus control sera. These data suggest that in achalasia more MMP-9, relative to MMP-2, is produced and that the produced levels of proMMP-9 seem to become more efficiently activated than in controls, which suggests a swinging of the protease balance towards proteolysis.

### Gelatinases in biopsies from achalasia patients

**Fig. 3:**
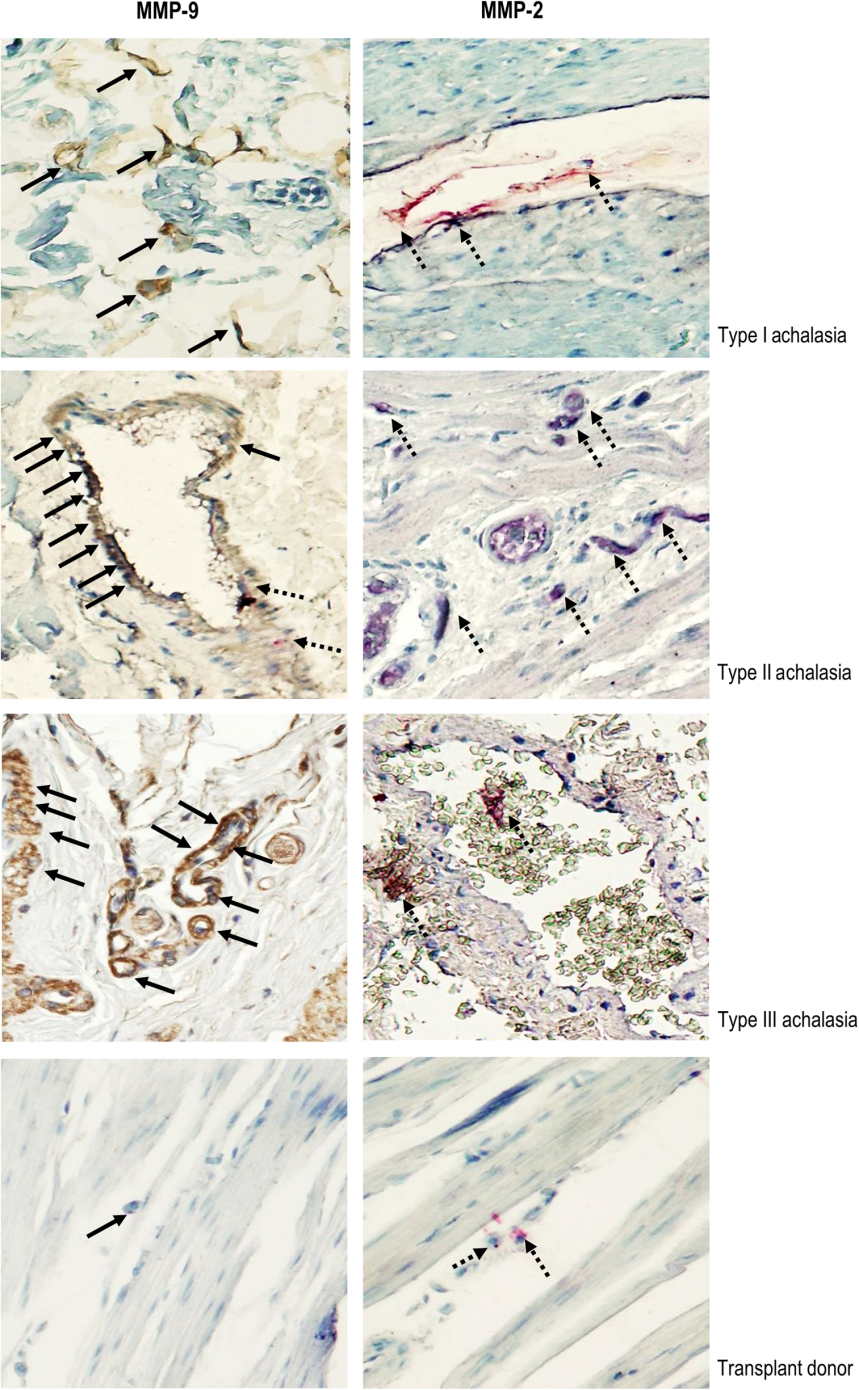
Immunohistochemical analysis of MMP-2 and MMP-9. Representative immunostaining of MMP-9 (left panel) and MMP-2 (right panel) in tissue biopsies from achalasia patients. Solid arrows depict MMP-9 expression (in brown). Dotted arrows show MMP-2 expression (in red). Original magnification was X320. Below the achalasia tissue samples, a normal biopsy from the esophagus of a transplant donor is included. The immunoreactivity for MMP-9 was with the monoclonal antibody REGA-2D9, whereas the immunoreactivity for MMP-2 was with a polyclonal antibody

**Fig. 4:**
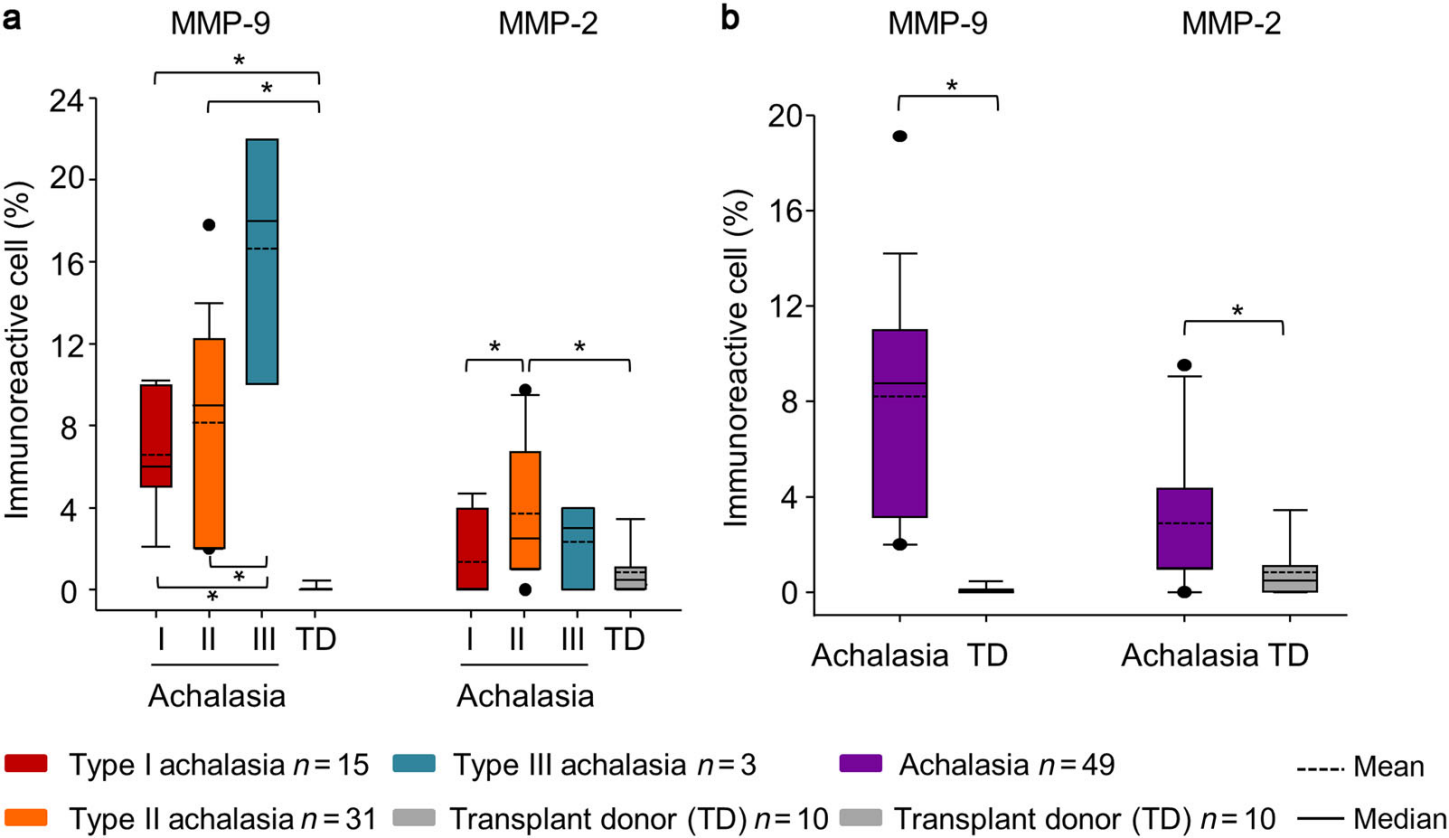
Morphometrical analysis of immunoreactivities for MMP-9 and MMP-2. **a** Percentages of immunoreactive cells per microscopic field for MMP-9 and for MMP-2 in type I, II, and III achalasia patients and esophageal tissue of transplant donors. **b** Overall tissue expression of MMP-9 and MMP-2 in an achalasia cohort. The results are expressed as mean (horizontal dotted line), median (solid line), and 5th/95th percentiles of MMP-9^+^ and MMP-2^+^ cells. Transplant donors (TD) were included as controls. *p < 0.05. Numbers of analyzed biopsies are provided together with the color codes

The increased serum levels of gelatinases prompted us to investigate achalasia tissues as a possible source of MMP-2 and MMP-9. Demographic, clinical and laboratory variables were comparable for the four investigated patient groups (Table 1). We investigated by immunohistochemical analysis the presence of MMP-2 and of MMP-9 in all biopsies. Figure 3 exemplifies prototypic data from this analysis. MMP-2‒expressing cells were mainly associated with perivascular inflammatory infiltrates and to a lesser extent to the myenteric plexus. MMP-9‒producing cells were largely associated to the parenchymal cells and the myenteric plexus. An extensive damage to the plexus was determined in those tissues with a higher number of MMP-9^+^ cells. It was important to highlight that the MMP-9‒producing cells were different from those that synthesized MMP-2. These data were refined with a detailed immunohistomorphometric analysis. The percentages of MMP-9‒expressing cells were significantly higher in patients with achalasia when compared with those producing MMP-2 (Table 1). Type III achalasia patients showed increased expression of MMP-9, followed by type II, type I and transplant donors. The statistical analyses, comparing the types of achalasia and discriminating the total cohort of achalasia patients versus the control group are provided in the histograms in Fig. 4a, b, respectively. From this analysis, it was clear that both gelatinases, MMP-2 and MMP-9, are associated with local events in esophageal tissues. In particular, MMP-9 immunoreactivity increased significantly according to type of achalasia. This was clear from multiple comparisons versus the control group of normal transplant donors with the use of Dunn’s method, as well as by pairwise multiple comparison procedures with the Holm-Sidak method. It needs to be noticed that the cohorts of type III achalasia patients (*n* = 3) and the transplant donors used as controls (*n* = 10) were rather small. However, the parameter variabilities were considerably larger in the type III achalasia patients versus the control subjects. For these reasons, the achalasia type III cohort is provided for reference only, whereas the control group of transplant donor biopsies was representative. Collectively, the above data formed a solid basis to test the remnant epitope REGA-paradigm in achalasia, i.e. to evaluate whether autoantigens in achalasia are substrates of MMP-9.

### MMP substrates in achalasia

**Fig. 5:**
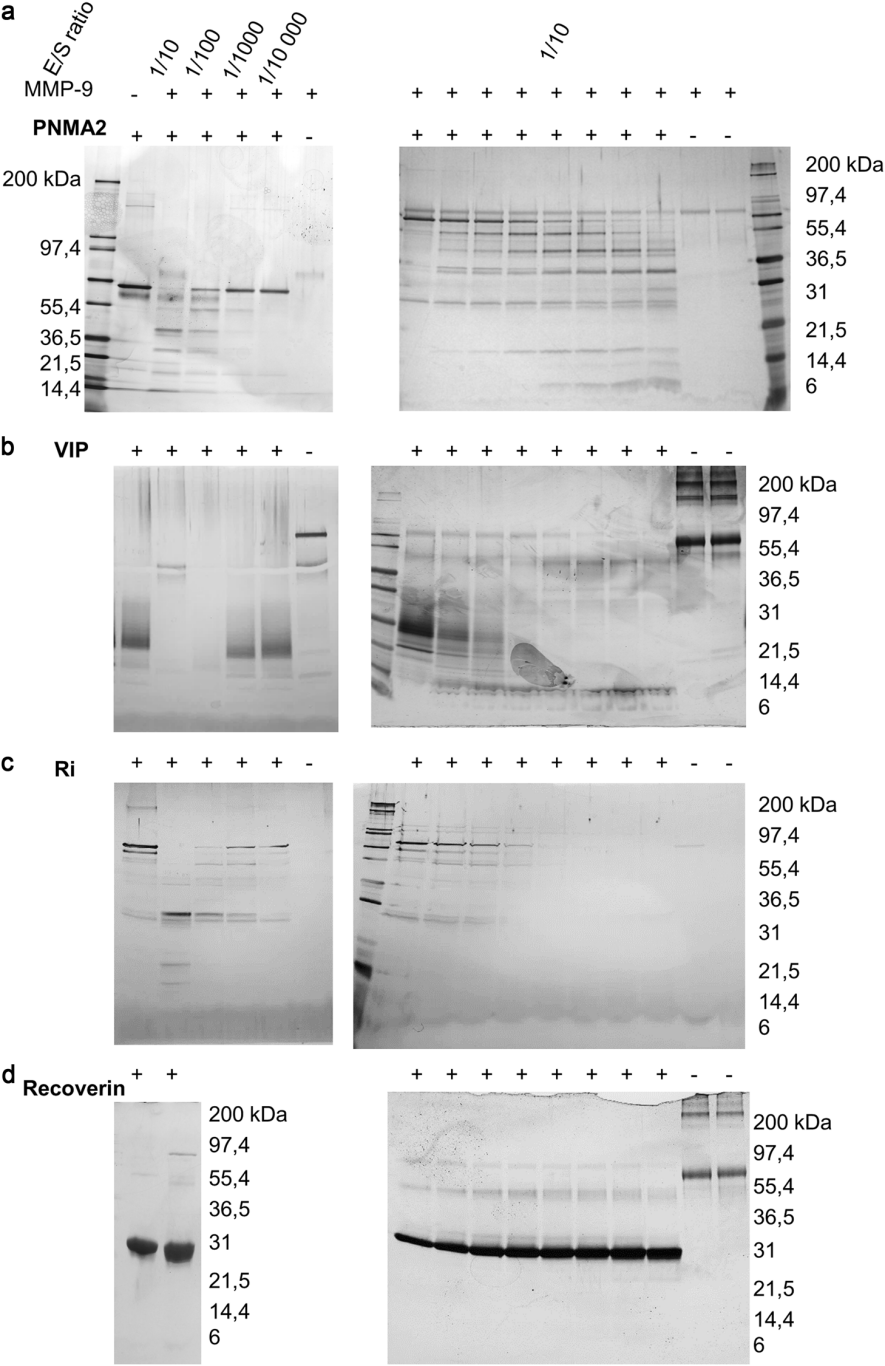
Autoantigenic substrate cleavage by MMP-9. **a** Left: incubation of PNMA2 (1 µM) alone or with active MMP-9 at different enzyme/substrate (E/S) ratio’s (1/10, 1/100, 1/1000, and 1/10 000) overnight at 37 °C, shows cleavage into PNMA2 fragments after SDS-PAGE separation and silver staining. Right: incubation of PNMA2 with active MMP-9 (E/S ratio of 1/10) for 0, 5, 10, 30, 90, 180, 420 min and overnight. **b** Similar experimental set up for VIP (10 µM), **c** Similar experimental set up for Ri (0.2 µM). **d** Recoverin (10 µM) was not cleaved after prolonged incubation at an enzyme/substrate ration of 1/10. For all panels molecular weight standardization was included and the molecular weight marker proteins are indicated in kilodaltons (kDa). All photographs are aligned in such ways that for all cleavage experiments the conditions were similar to the ones indicated in the top photographs of panel A

To study the possible role of MMP-9 in generating autoantigenic remnant epitopes, we first listed all known autoantigenic proteins in achalasia (Table 2). These were defined on the basis of the presence of autoantibodies in patients^29^ (amphiphysin, CV2, PNMA2 (or Ma-2/Ta), the onconeural antigens Ri, Yo, and Hu, recoverin, SOX-1, Titin, GAD65) or because of observed reduced levels in achalasia (VIP, nNOS, S-100 protein, substance P, PGP 9.5). Commercially available proteins were first controlled for purity and quality and next incubated with active MMP-9 at different enzyme/substrate ratios and for several incubation times. The activity of the enzyme preparations was monitored by using actin as a known substrate of MMP-9. Several of the listed autoantigens were already known to be cleaved by MMP-9 (Table 2 and accompanying references). Here, we focused on the discovery of novel MMP-9 substrates and in Fig. 5 we illustrated that PNMA2, VIP, and Ri are cleaved into remnant epitopes by MMP-9. We thus defined several new substrates of MMP-9 and added these to the list of achalasia autoantigens. We summarized all the previous and new data in Table 2 and provided an overview of all present degradome data for MMP-9 in achalasia. Specifically, S100A8 and S100A9^30^ and substance P^31^ have previously been described as substrates of MMP-9. GAD65, a common autoantigen in type 1 diabetes, was previously described by our group as a substrate of MMP-9 (Supplemental Fig. 3, from Doctoral Thesis by Francis J. Descamps, University of Leuven 2005). Recoverin was not a substrate of MMP-9 (Table 2 and Fig. 5). In conclusion, gelatinase B cleaves a considerable number of achalasia autoantigens into remnant epitopes.

**Table 2:** Known and novel autoantigen substrates of MMP-9 in achalasia

	Reduced levels	Auto-antibodies	MMP-9 substrate (known reference)	MMP-9 substrate (new)
VIP	+			✓
nNOS	+			
S-100	+		+A8/A9(30)	
Substance P	+		+(31)	
PGP 9.5	+			
Amphiphysin		+		
CV2		+		
PNMA2		+		✓
Ri		+		✓
Yo		+		
Hu		+		
Recoverin		+		Not a substrate
SOX-1		+		
Titin		+		
GAD65		+		✓

Autoantigens in achalasia (column 1) are discriminated on the basis of disappearance (column 2) or presence of detectable autoantibodies (column 3) in patients with achalasia. Columns 4 and 5, respectively, document whether these autoantigens were already known or discovered in the present study as substrates of MMP-9

### Correlations in achalasia patients between gelatinase ratios and clinical variables

With the finding that gelatinases cleave a substantial number of achalasia autoantigens into remnant epitopes and gelatinase ratios may be of diagnostic value, we correlated clinical parameters with gelatinase ratios. We found a positive correlation in the achalasia group between the autoimmune disease comorbidity and the ratio of active/proMMP-9 (Spearman’s rho: 0.954, *P* = 0.006).

A positive correlation was determined in achalasia type III between tobacco consumption and active/proMMP-9 (Spearman’s rho: 0.828, *P* = 0.042); while a negative correlation was found between overweight and active/proMMP-9 (Spearman’s rho: −0.828, *P* = 0.042). Other correlations were not significant.

## Discussion

Whereas achalasia is suggested to be an autoimmune disease of the esophagus^32^ with major involvement of adaptive immune cells, we here emphasize the role of the innate immune system and of regulated extracellular proteolysis in generating remnant epitopes^11,12^. Indeed, in earlier studies the role of MHC II-mediated activation of T lymphocyte subsets and their cytokine profiles and the role of autoantibodies from B lymphocytes were studied in achalasia^3,4^. Here we studied possible steps that may occur before such adaptive immune activation, i.e. during the initiation phase of the disease^12^ or, alternatively, that may reinforce adaptive immune responses, i.e. by generating more processed autoantigens. Innate immune mechanisms have been less or not studied and may be equally important than the adaptive immune processes because (i) these may be easier to target than clonal adaptive immune responses; (ii) their targeting may prevent autoimmune boosting; (iii) interference at the innate level usually does not fade out with repeated interventions, whereas adaptive immune intervention may result in treatment resistance; (iv) innate immune intervention essentially leaves the adaptive immune system intact for the defense against and elimination of infections and (v) although such innate immune-based treatment may not be against an etiological agent, it would still constitute a mechanism-based therapy for achalasia.

Prior data^4^ indicated that it was worthwhile to investigate MMPs in sera as potential markers for achalasia, much in a similar way as we did for inflammatory bowel diseases^25^. Thanks to the development of standardized quantitative zymography^23^ we were able to execute a multiparametric analysis of all serum proteoforms of gelatinases. To our surprise, we found alterations in MMP-2 levels, rather than in the serum titers of MMP-9. MMP-2 is a constitutively expressed enzyme, because its gene has a rather simple promoter region with CIS elements for transcription factors yielding basal expression, whereas the promotor region of MMP-9 contains many inflammation-inducible elements^33^. Overall, basal MMP-2 levels were lower in achalasia in comparison with control levels and the ratio of MMP-9 versus MMP-2 was increased in achalasia. The changes in the latter parameter are in line with observations in other autoimmune diseases, including multiple sclerosis, diabetes and rheumatoid arthritis^12^. A second finding was a significant increase in activated MMP-9 in achalasia sera. Whereas it is difficult to speculate about the origin of the circulating MMP-9, the presence of many MMP-9-expressing cells in achalasia biopsies suggest at least a trigger from the local tissue. Anyhow, our detection and analysis of gelatinase forms in sera is interesting for biomarker development for achalasia, because serum analysis is simple in comparison with HRM or endoscopic biopsy and histopathology analysis. Future studies with other cohorts will be used to validate MMP analysis as a serum marker for achalasia and whether the levels of specific parameters or ratios correlate with the severity of the disease.

On the basis of higher zymolysis levels of activated MMP-9 in achalasia sera and biopsies, in situ expression was evaluated on a number of biopsy samples. In addition, normal tissues from transplant donors were included for comparisons. From this analysis, it was clear that (i) MMP-9 expression was locally more important than MMP-2 expression (ii) the cells expressing MMP-9 were different from those that expressed MMP-2 (iii) the level of local MMP-9 expression increased with the severity of the disease, as typed according to the Chicago classification. Conclusions should be made with caution due to the limited achalasia type III patient numbers (zymography on sera: *n* = 5 and tissue analysis: *n* = 3). Moreover, current clinicopathologic findings on subtyping of achalasia suggest that type I achalasia represents a more advanced stage that potentially progressed from type II achalasia and that type III could represent a different disease^4,34,35^. Our findings suggest that the gelatinases play a role during the initial stage of the disease and remain detectable in the serum during disease progression. We further strengthened the evidence that these gelatinases, detected by immunoreactivity, were indeed catalytically active, i.e. not only in the inactive proform and not inhibited by natural inhibitors. As a logical consequence, we next studied whether MMP-9 might contribute to autoantigen processing into remnant epitopes. For this, a comprehensive list was made of all known achalasia autoantigens (Table 2) and these molecules were evaluated as known or novel substrates of MMP-9.

Besides comparing achalasia patients to healthy controls, a small group of patients with an Esophagogastric Junction Outflow Obstruction (EJOO) having elevated median IRP (>15 mm Hg) with sufficient evidence of peristalsis was included in this study as an extra control (*n* = 7, data not shown). On zymography analysis, EJOO patients had lower MMP-9 levels compared to both achalasia patients and healthy controls and lower MMP-2 levels compared to controls. Ratios of MMP-9/MMP-2 and act/proform MMP-9 of EJOO patients were similar as observed in healthy controls, in contrast to the significant increase observed in achalasia patients versus healthy controls.

S100A8 and A9^30^ and substance P^31^ were already known substrates of MMP-9 (Table 2). Here, the documentation of PNMA2, Ri, VIP, and GAD65 as novel substrates of MMP-9 adds information to the paradigm that extracellular proteolysis might play a role in the pathogenesis of organ-specific autoimmune diseases^11,12^.

In conclusion, one can view achalasia as an autoimmune disease against the neuromuscular junction of the myenteric plexus. In this disease, the extracellular proteolysis-paradigm seems a valid addition to understand the pathogenesis. Indeed, before classical intracellular antigen processing and presentation of autoantigens in MHCII happens, innate immune mechanisms triggered by environmental stimuli lead to local extracellular proteolysis in the myenteric plexus of the esophagus and the cleavage of intact autoantigenic proteins into remnant epitopes. Consequently, this mechanism provides an excess of peptides for classical antigen presentation and (re)activation of T helper cells and B cells. In addition, we define serum markers and suggest that MMP-9 inhibitors, such as tetracyclines^19,36,37^ or immunosuppressive drugs for myeloid cells, may be worthwhile to test as adjuvant treatments for achalasia.

## Study Highlights

### What is the current knowledge

- Achalasia is an esophageal motility disorder most likely due to an autoimmune response.
- Diagnosis of achalasia is based on invasive manometry and endoscopy.
- So far, therapeutic options are limited to myotomy and endoscopic pneumodilatation.

### What is new here

- Serum levels of MMP-9/MMP-2 and activated MMP-9/proMMP-9 are increased in achalasia patients.
- High MMP-9 expression in situ is associated with extensive plexus damage.
- MMP-9 may contribute to the pathogenesis by cleaving achalasia-associated autoantigens into remnant epitopes generating autoimmunity (REGA paradigm).

### Translational impact

- The analysis of MMP-2 and MMP-9 proteoforms and the ratios of MMP-9/MMP-2 and actMMP-9/proMMP-9 in serum may become valuable tools for achalasia diagnosis and prognosis.
- In view of achalasia autoantigen processing into remnant epitopes, probably at sites of plexus damage, it is worthwhile to test MMP-9 inhibition, e.g. by tetracyclines, as treatment for achalasia.

## Electronic supplementary material

Supplemental Figure 1

Supplemental Figure 2

Supplemental Figure 3

Supplemental Figure 4
