# Supplementary figures and images for: Gelatinase B/Matrix Metalloproteinase-9 as Innate Immune Effector Molecule in Achalasia
Source: Clin Transl Gastroenterol. 2018 Nov 19;9(11):208. doi: 10.1038/s41424-018-0076-6 (PMC6240577; doi:10.1038/s41424-018-0076-6)

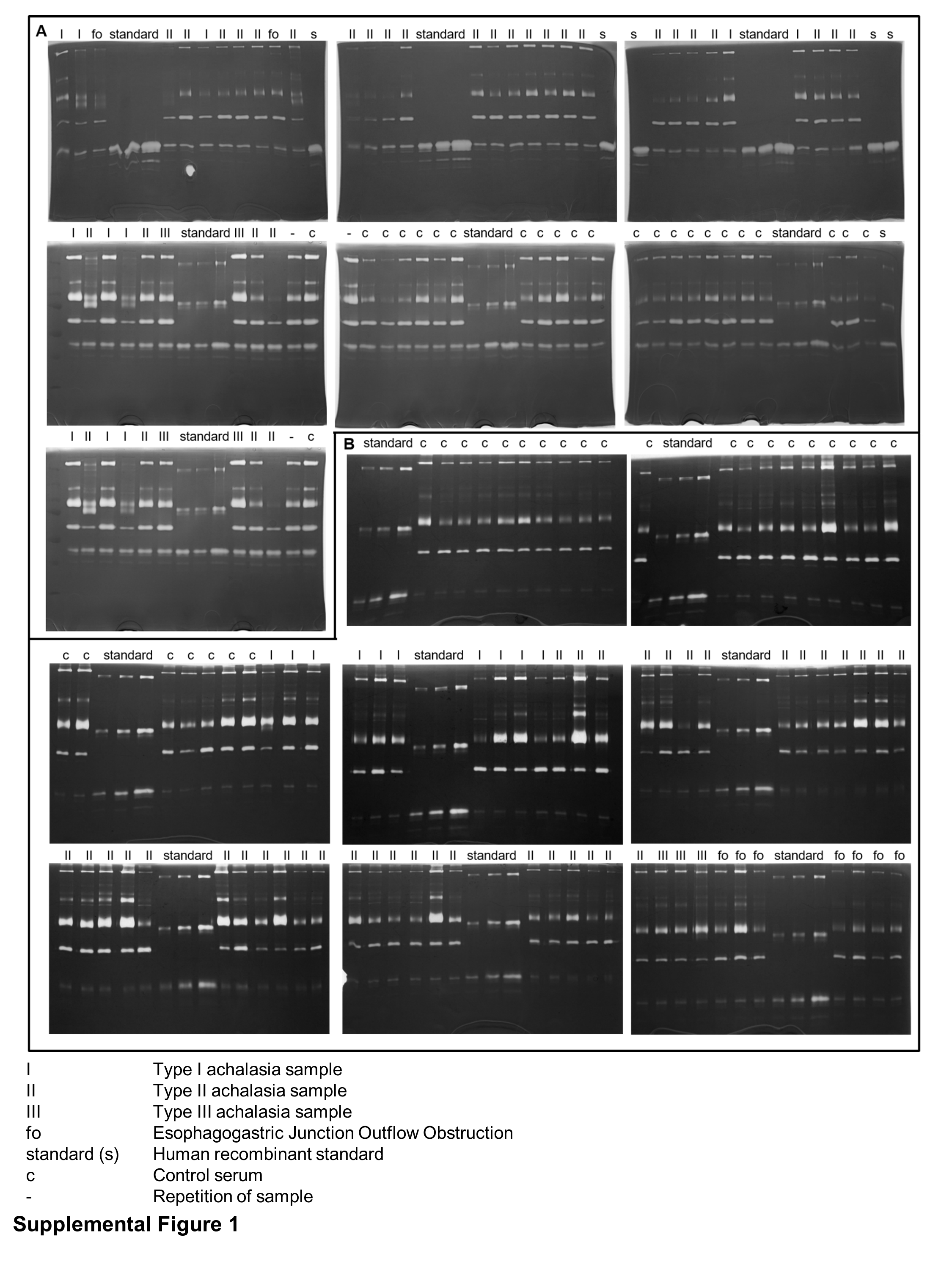

Supplement: Supplementary file 1 — Supplemental Figure 1 [file 41424_2018_76_MOESM1_ESM.tif]

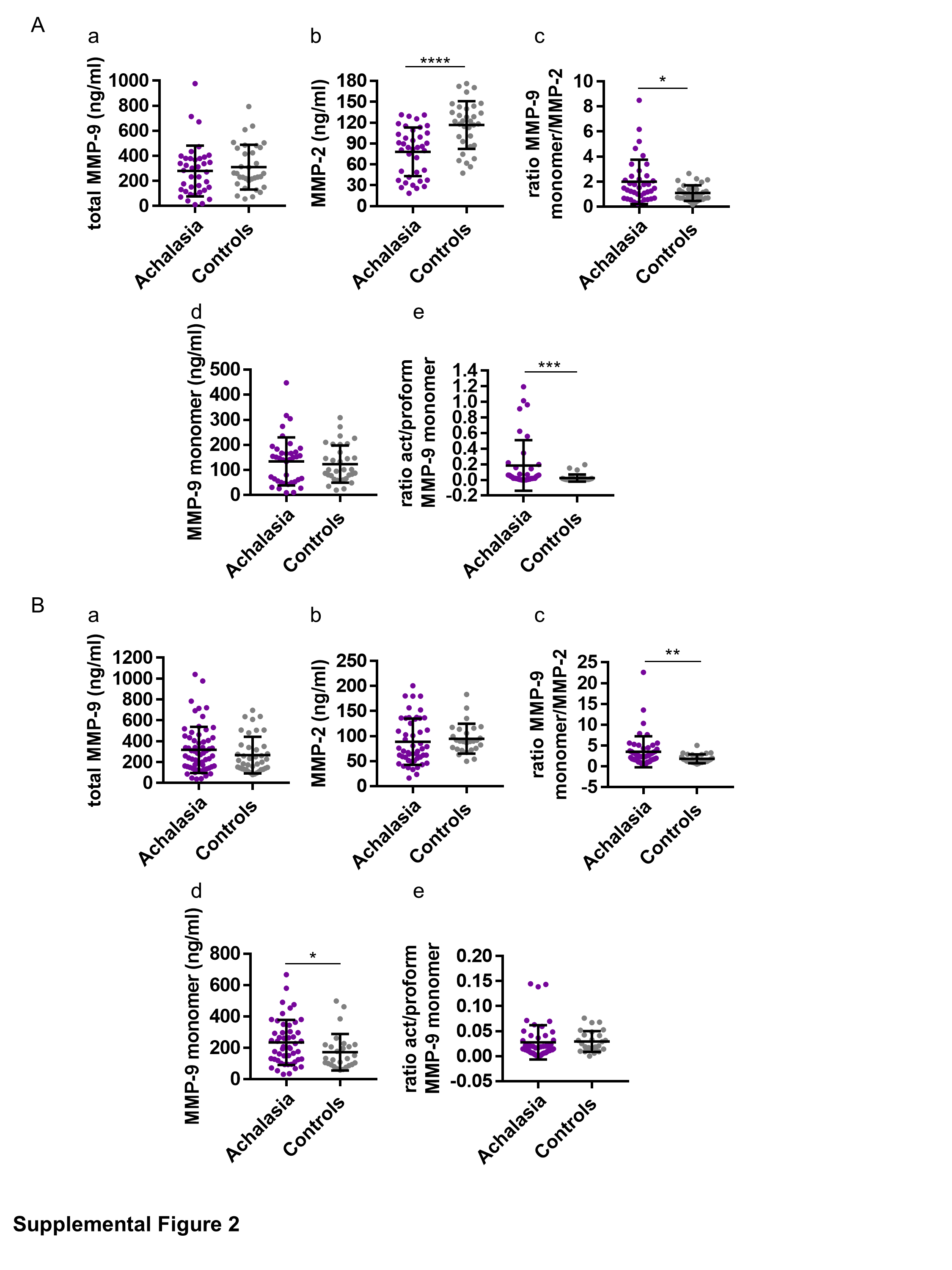

Supplement: Supplementary file 2 — Supplemental Figure 2 [file 41424_2018_76_MOESM2_ESM.tif]

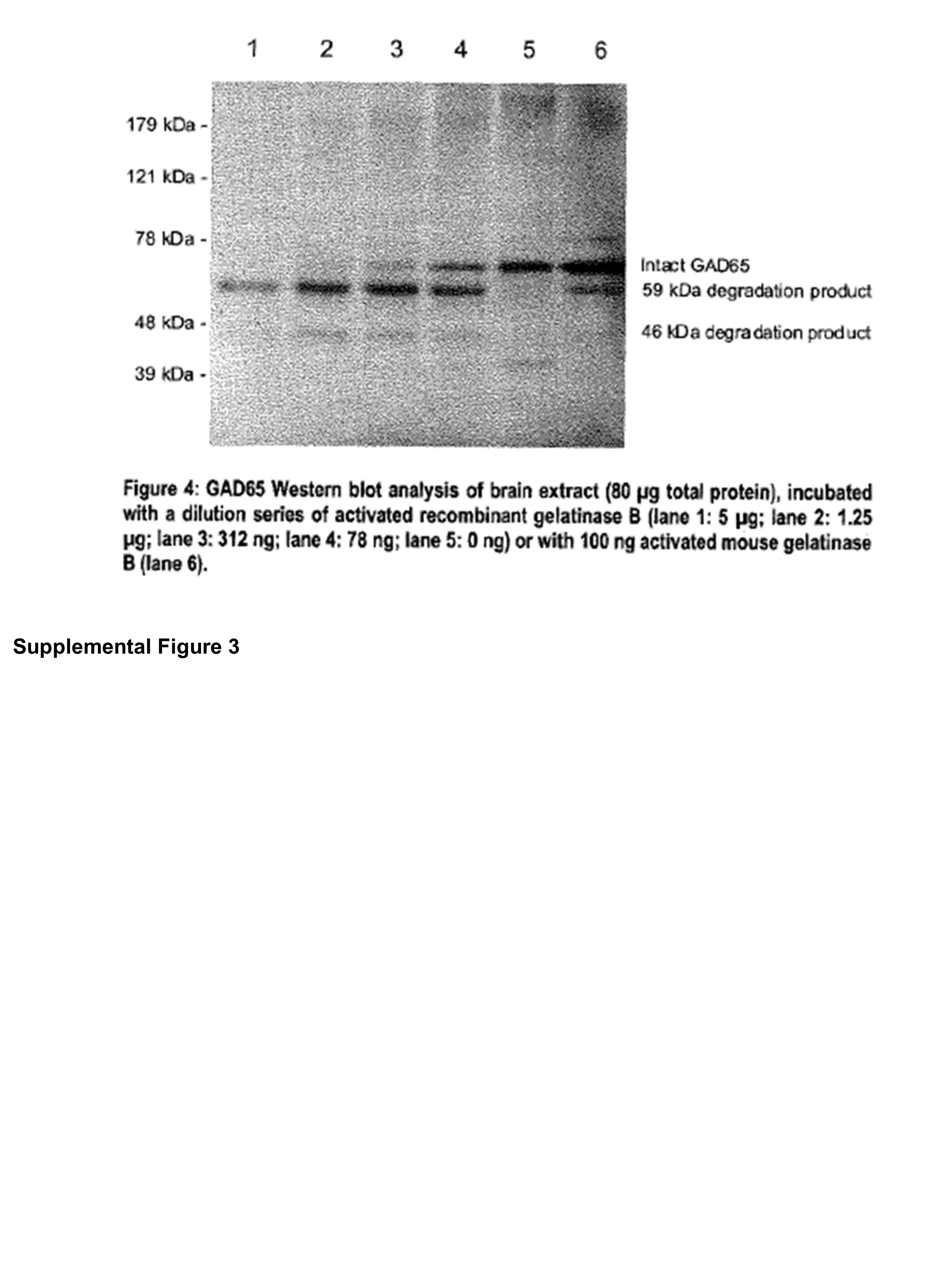

Supplement: Supplementary file 4 — Supplemental Figure 4 [file 41424_2018_76_MOESM4_ESM.tif]
