# Supplementary material for: Gelatinase B/Matrix Metalloproteinase-9 as Innate Immune Effector Molecule in Achalasia
Source: Clin Transl Gastroenterol. 2018 Nov 19;9(11):208. doi: 10.1038/s41424-018-0076-6 (PMC6240577; doi:10.1038/s41424-018-0076-6)
Supplement: Supplementary file 3 — Supplemental Figure 3 [file 41424_2018_76_MOESM3_ESM.docx]

**Supplemental Figure 1.** Zymography gels of serum samples of patients and controls of A. the first cohort and of B. the second cohort.

**Supplemental Figure 2.** Quantitative gelatin zymography analysis of achalasia patient sera of two independent cohorts.

A. Quantitative gelatin zymography results of achalasia (n=38) and controls (n=33) serum samples of the first cohort 1. B. Quantitative gelatin zymography results of achalasia (n=50) and healthy controls (n=27) serum samples of the second cohort. a. Levels of total MMP-9 in achalasia *versus* control sera. b. Levels of MMP-2 in achalasia *versus* control. c. Ratios of MMP-9 monomers and MMP-2 in the comparisons of sera from achalasia patients and controls. d. Levels of monomer forms of MMP-9. e. Ratios of monomeric activated MMP-9 and proMMP-9 in the comparisons of achalasia and control sera. For all groups the mean levels +/- SD are provided, * indicates p<0.05; ** indicates p<0.01; *** indicates p<0.001 and **** indicates p<0.0001.

**Supplemental Figure 3.** Proteolytic cleavage of GAD65 by MMP-9.

Whole brain extract from C57BL-6 mice was prepared and aliquots of 80 µg were incubated with recombinant human MMP-9 and separated by SDS-PAGE. The proteins were transferred to a solid support and reacted with an antibody against mouse GAD65.

Source: PhD dissertation, Francis Descamps, KU Leuven 2005, page 75.
